# Theoretical Threshold for Estimating the Impact of Ventilation on Materials’ Emissions

**DOI:** 10.1021/acs.est.3c09815

**Published:** 2024-03-06

**Authors:** Fredrik Domhagen, Sarka Langer, Angela Sasic Kalagasidis

**Affiliations:** † Department of Architecture and Civil Engineering, Chalmers University of Technology, SE-41296 Gothenburg, Sweden; ‡ IVL Swedish Environmental Research Institute, P.O. Box 53021, SE-40014 Gothenburg, Sweden

**Keywords:** ventilation, VOC, emission rate, material emission, indoor air quality, off-gassing

## Abstract

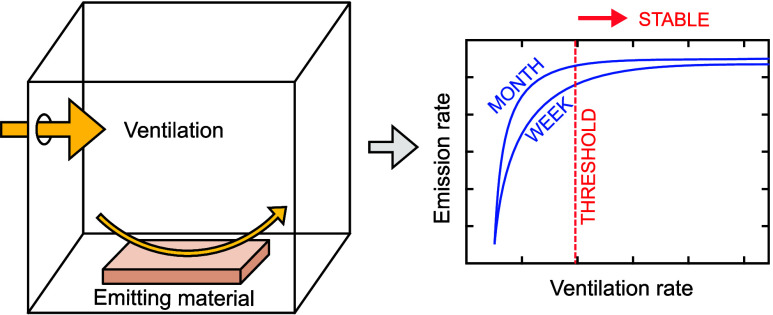

In new buildings, nonoccupant VOC emissions are initially high but typically decrease within months. Increased ventilation is commonly used to improve indoor air quality, assuming it speeds up VOC off-gassing from materials. However, previous research presents inconsistent results. This review introduces a simplified analytical model to understand the ventilation–emission relationship. By combining factors such as diffusivity, emitting area, and time, the model suggests the existence of a theoretical ventilation threshold beyond which enhanced ventilation has no further influence on emission rates. A threshold of approximately 0.13 L s^–1^ m^–2^ emitting area has been found for various VOCs documented in the existing literature, with which the conflicting results are explained. It is also shown that the threshold remains notably consistent across different boundary conditions and model resolutions, indicating its suitability for real-world applications.

## Abstract 2

Prior research found mixed results regarding the impact of increased ventilation on VOC emission rates from materials. This study introduces an analytical method to identify when increased ventilation does not lead to higher material emission rates.

## Introduction

Volatile organic compounds (VOCs) belong to the most abundant group of pollutants found in the indoor air.^1−3^ VOCs are often perceived by occupants as malodor^4^ and may have negative effects on mental performance and productivity.^5−7^ In addition, the levels of non-occupant-related VOCs, that are emitted from materials, are usually higher in new buildings, and it may take several months before they decline to acceptable levels.^8−10^ This decline is a nonlinear process influenced by both diffusion within emitting materials and a room’s ventilation.^11^

A common strategy for dealing with high VOC levels in new buildings is to arbitrarily increase ventilation flow rates. This serves two goals: to reduce VOC concentrations in indoor air to acceptable levels^12^ and to increase material emission rates for faster depletion of the emission sources.^13^ For example, building certification systems like LEED^14^ and WELL^15^ promote increasing ventilation rates above existing ventilation standards during initial stages of a building’s occupancy. In LEED, a flush-out (excessive ventilation) before occupancy is also awarded credits and promoted as an alternative to indoor air quality testing.

Another method proposed in the literature involves excessive heating known as “bake-off” or “bake-out” to enhance off-gassing. This method intensifies emissions from materials by first raising the indoor temperature to 30–40 °C for up to a week at standard ventilation, followed by returning to normal heating and intensified ventilation to remove emitted VOCs.^16,17^

Unfortunately, there is uncertainty regarding whether the building’s heating system can achieve the necessary temperature levels in all materials and items within the room for enhanced off-gassing. Moreover, it results in increased energy consumption and costs due to excessive heating and ventilation. These are likely the reasons why the bake-off method is not commonly used in northern countries like Sweden.

Excessive ventilation in new buildings is therefore a common method to handle early stage emissions. However, previous research shows inconsistent results regarding the impact of enhanced ventilation on materials’ emission rates. For example, Gunnarsen^18^ measured emission and ventilation rates for several materials in a test chamber and concluded that emission rates correlated with the ventilation at low rates but not at higher rates. According to the author, at higher ventilation rates, emissions are limited by the materials’ diffusivity, while at lower rates, it is the ventilation that affects the emission rate. It is then reasonable to increase the ventilation rate in order to shorten the off-gassing time to the level where emission rates have stabilized. This explanation is also supported by Brown and Wolkoff.^8,19^ Other studies reported a dependency between emission and ventilation rates.^20−22^

Conversely, a recent review by Hølos et al.^13^ draws a conclusion that emission rates are not noticeably accelerated by increased ventilation rate, at least not on the time scale of weeks or months. The review found only one field study, Hodgson et al.,^23^ in which the emission rate is increased along with the increased ventilation rate. Ye et al.^24^ raises concerns about excessive ventilation. Their proposed procedure for determining ventilation rates in new buildings involves identifying a leading substance, which has the highest concentration compared to a reference or guideline value. By combining the initial concentration of the leading substance, the diffusion coefficient of the material, and its emitting area, they calculate the required air change rate and time for a complete off-gassing of the materials. They show that the latter is approximately 2.5–5 years long. While uncertainty in material data is a major challenge with this approach, the study highlights potential risks of overventilation if ventilation rates are not adjusted in accordance with emission rates.

For the purpose of clarifying the inconsistent results reported in the literature, we introduce, in this article, a simplified analytical model to understand the ventilation–emission relationship. Advanced and data intensive models, such as those by Xiong et al.,^25^ Liu et al.,^26^ Deng et al.,^27^ and Zhang et al.,^28^ focus on characterizing and predicting material emissions using compound’s initial concentration, *c*_0_, diffusion coefficient, *D*_m_, and material–air partition coefficient, *K*_ma_, in controlled laboratory settings. Unlike these models,^25,26^ the simplifications used here offer a straightforward approach to identify when increased ventilation remains effective, a ventilation threshold. Therefore, in comparison to the analytical models provided in the literature, our approach differs in the scope and input data needed for analysis. Those models apply to materials with known thickness and surface transfer coefficients, which, as we argue, complicate practical applications when the goal is to dimension the ventilation air flow for off-gassing purposes. For example, in the section Verification of the Analytical Model we demonstrate that surface transfer coefficients have negligible impacts on the ventilation threshold. We also motivate the simplification of treating each material as a semi-infinite region, which results in a slight overestimation of the ventilation threshold. The overestimation is reasonable given that the ventilation threshold is inherently an approximation.

The novelty of our research lies in featuring the concept of the ventilation threshold, which is a new way of understanding the relation between material emission rates and ventilation rates and not in the development of a simplified analytical model itself. The unique features of the proposed model can be summarized as (1) introducing the concept of a ventilation threshold, (2) providing an analytical, general model for examining this relationship in terms of an emission time-constant (*t*_c_), and (3) making an initial attempt to quantify this threshold.

## Methods

### Analytical Model

The mass transfer of VOC between porous materials and indoor air depends on several physical processes such as boundary diffusion, diffusion within materials, and sorption at the interface between the solid surfaces and air (both at the material surface facing the room and at pore surfaces inside the material).^29^ Furthermore, the sorption involves a microscopic mass transfer between a solid surface and its near surrounding air and depends on the nature of the adsorbate and adsorbent.^29^

Our goal is to investigate the relation between ventilation and the emission rate under typical building operation conditions. This means that elevated temperatures, such as those during bake-off, are not considered. Also, as observed in earlier studies, typical, small indoor variations in air temperature have negligible effects on emission rates.^12^

There are several mathematical models that account for both diffusion within materials and sorption on surfaces.^30^ The model proposed here is based on the commonly used *c*_0_–*K*–*D* models.^25,26,31−34^ These models describe the one-dimensional diffusion of VOCs through a dry and homogeneous material at a macroscopic level. Necessary relations for diffusion and retention of VOCs are then typically derived by analogy with the transient moisture transport in porous media. Due to these simplifications, a coupling between the diffusion of VOCs in materials and their convection to/from the surrounding air is conveniently established. Because the focus of this work is on determining ventilation thresholds rather than on predicting emissions, the cited modeling approach is found to be convenient and adopted.

**Figure 1:**
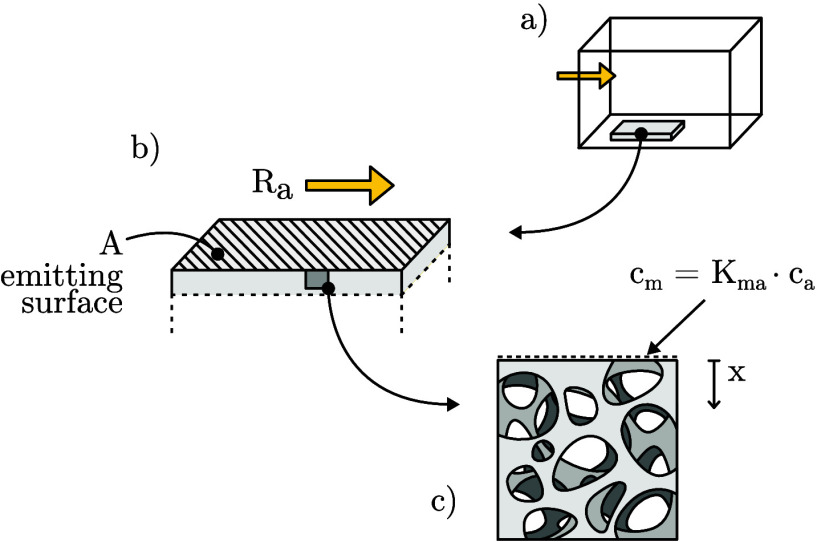
Graphical representation of the model: (a) material placed inside a ventilated room, (b) emitting surface area, (c) internal surface area and interface between material and air.

To facilitate the derivation of the analytical model, several simplifications have been made. The emitting material is assumed to be homogeneous and semi-infinite, thus neglecting the impact of boundary conditions other than those present at the emitting surface (see Figure 1). This assumption allows for disregarding any uncertainty in the material thickness. Since the purpose of the model is to estimate emissions from materials at early stages of new buildings, the semi-infinite material is a good representation as long as the diffusion process has not affected the backside of the material. For thin materials and long time spans, this simplification leads to an overestimated emission rate, which approaches a theoretically maximal one (see Figure 3). Furthermore, diffusion of VOCs within the material is assumed to be one-dimensional in the direction normal to the emitting surface. Also, we assume that each VOC emitted from the material is well-mixed with the air in the room. The VOC concentration in the room can therefore be understood as a spatially averaged concentration that varies only with time.

The one-dimensional diffusion of VOCs, including sorption, in a homogeneous material is described by Fick’s second law:
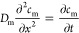
1The coordinate axis, *x*, points inward from the surface of the material, *D*_m_ (m^2^ s^–1^) is the diffusion coefficient for the material, and *c*_m_ (μg m^–3^) is the concentration in the material.

The concentration of absorbed VOCs in the material (material phase), *c*_m_ (μg m^–3^), generally differs from the concentration of VOCs in its air pores (air phase), *c*_a_ (μg m^–3^). By assuming an equilibrium between them, they can be linked by the dimensionless partition coefficient, *K*_ma_:
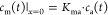
2

For simplicity, we assume the same initial concentration for both the material and indoor air. In reality, the initial concentration in indoor air is likely close to that in the supply air. However, this assumption facilitates derivation of the model, and as will be shown later, it affects the concentration only at times close to zero.

The initial concentration is denoted as *c*_0_ (μg m^–3^):
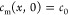
3

**Figure 2:**
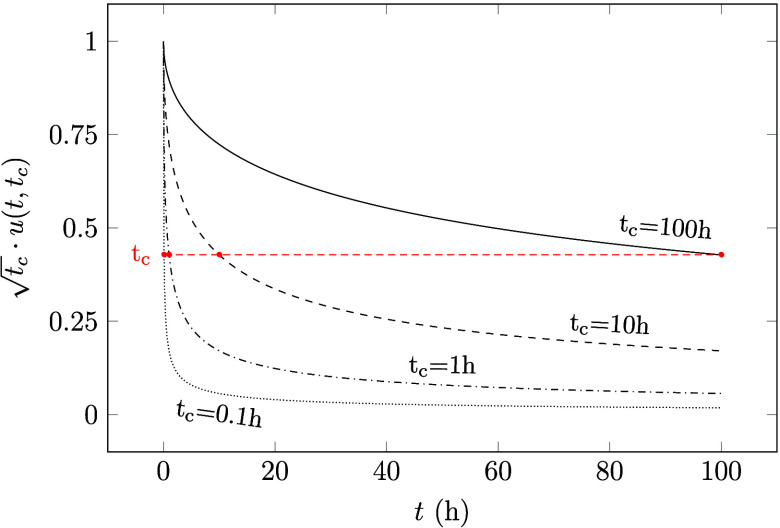
plotted against *t* for different time constants, *t*_c_.

The mass balance across the material-air interface is described with the well-known Robin boundary condition, sometimes referred to as the mixed boundary condition.^35−37^ Here it couples the mass transport by diffusion and ventilation air:
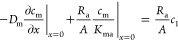
4Here, *c*_1_ (μg m^–3^) is the concentration in the supply air, *R*_a_ (m^3^ s^–1^) is the ventilation rate in the room, and *A* (m^2^) is the surface area of the emitting material or emitting area. We assume that the concentration at an infinite depth equals the initial condition:
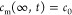
5The solution for the differential equation described above with the given boundary and initial conditions is found by utilizing Laplace transformations and reads
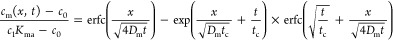
6The coefficient, *t*_c_ (s), is a time constant that describes the relation between emitting area, diffusivity, and ventilation rate:
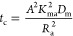
7A small *t*_c_, caused by, for example, high ventilation rates, means that the emission rate in the room decreases quickly over time; see Figure 2. The surface concentration in the material phase depends only on time:
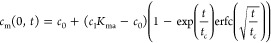
8Since we assume a well-mixed air, the concentration in the room is
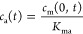
9In particular, the emission rate, *E* (μg s^–1^), at the surface of the material toward the room, , becomes
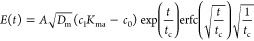
10or
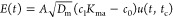
11where *u* denotes the change in emission rate over time:
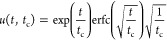
12The function *u* has the following limit as *t*_c_^–1^ approaches infinity:^38^
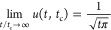
13Figure 2 shows *u* plotted against time (*x*-axis) with the time constant as parameter taking the values of *t*_c_ = 0.1, 1, 10, and 100 h. Also, the product  is the same whenever *t* = *t*_c_ (red dashed line).

### Correlation between Ventilation and Emission Rate

**Figure 3:**
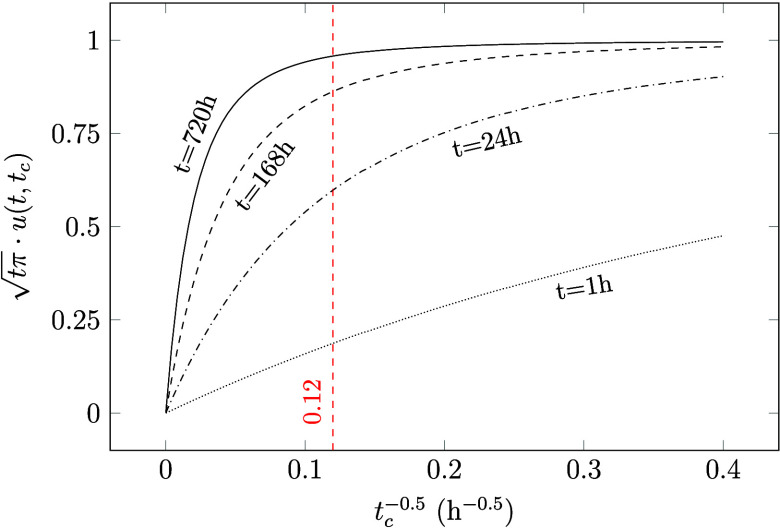
plotted against  as it approaches its limit value  for different time scales, *t*.

The time constant, *t*_c_, is useful for understanding how the emission rate changes as either ventilation rate, emitting area, or diffusivity changes. In Figure 3, *t*_c_^–0.5^ is plotted against its maximum emission rate (that depends on the time, *t*), for four different time scales, 1 h, 24 h, 168 h (1 week), and 720 h (1 month). Plotting time with *t*_c_^–0.5^ as a variable (on the *x*-axis) rather than *t*_c_ simplifies the interpretation of the graph, making apparent, for instance, what happens if the ventilation rate is doubled. Here, doubling the ventilation rate corresponds to doubling the square root of *t*_c_^–0.5^.

From Figure 3 we can see that the emission rate changes rapidly at low values of *t*_c_^–0.5^, while it is steadier at higher values. There is also a noticeable difference between the curves plotted for shorter and longer time periods. For shorter times, for example, 24 h, there is still a change in emission rate even at higher values of *t*_c_^–0.5^. Conversely, increasing *t*_c_^–0.5^ above, for example, 0.12, has no significant effect on the emission rate for time periods of weeks to months. The reason is that, as time passes, more easily accessible VOC, near the surface, are depleted and the diffusion within the material becomes more dominating.

It is noteworthy that the relation between emission rates and ventilation rates (or *t*_c_) described by the curves in Figure 3 depends on the chosen time-scale, *t*, and the time-period, *t*_c_, but not on the initial concentration, *c*_0_, and the concentration in the supply air, *c*_1_.

### Physics-Based Threshold: An Example

Usually there is a time-lapse between installing materials and components in a new building and before the occupancy of the building starts. In the following example we assume that the time-lapse is 1 week.

In Figure 3 we can see that after 1 week (168 h), emission rates begin to stabilize for *t*_c_^–0.5^ higher than 0.12, marked with the vertical dashed red line. For week-long and month-long time scales, the emission rates at *t*_c_^–0.5^ = 0.12 are 86% and 96% of their theoretical values, respectively. This means that for ventilation rates that yield values of *t*_c_^–0.5^ lower than 0.12, increasing the ventilation rate would increase emissions, while at values above 0.12, increasing the ventilation rate will have little impact on the emission rate. Note that these % values are qualitatively determined as being close enough to the theoretical limit, while alternative, close enough values can also be used. The time constant *t*_c_ depends also on the material properties and the emitting area. By defining a most critical case in terms of material properties, we can, based on eq 7, derive a ventilation rate per emitting area. This ventilation is then a threshold for the considered emitting surface and is valid from 1 week after the material is installed.

By identifying a material with the lowest *t*_c_^–1^ value within the room, a theoretical upper limit for when an increased ventilation rate does not contribute to an increased emission rate can be estimated. The product *K*_ma_^2^*D*_m_ is of particular interest since it is decisive for the determination of *t*_c_; see eq 7.

**Figure 4:**
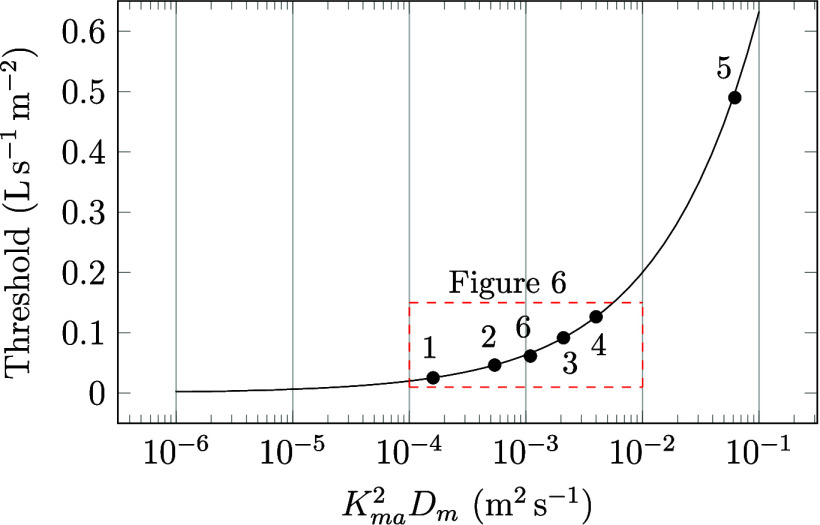
Ventilation thresholds calculated with eq 7 for *t*_c_^–0.5^ = 0.12 and plotted against *K*_m_^2^*D*_m_. Also, a selection of VOCs and materials (from Table 1) are marked in the graph: (1) toluene in carpet backing,^39^ (2) terpenes in particle board, (3) dodecane in gypsum board, (4) benzaldehyde in gypsum board, (5) formaldehyde in particle board,^40^ (6) nonane in carpet.^32^ The dashed rectangle marks the zoomed in part shown in Figure 6.

To illustrate this procedure, Table 1 shows a selection of materials with the given diffusion coefficients and partition coefficients. Benzaldehyde in the gypsum board is used to calculate the ventilation threshold because it yields one of the highest values for *K*_ma_^2^*D*_m_ ≈ 4 × 10^–3^, slightly higher than the value for α-pinene in the particle board. The ventilation threshold then becomes *R*_a_/*A* = 0.13l s^–1^ m^–2^ (per square meter emitting area), eq 7, for *t*_c_^–0.5^ = 0.12. Note that the calculated threshold depends on how *t*_c_^–1^ is chosen in Figure 3 and also on the material properties *K*_ma_ and *D*_m_. Different values for *t*_c_^–1^ can be chosen if needed. The effect of material properties can be seen in Figure 4, where the ventilation thresholds are plotted against *K*_ma_^2^*D*_m_ with *t*_c_^–0.5^ = 0.12.

**Table 1:** Diffusion Coefficients and Partition Coefficients for Several Materials and VOCs

	*D*_m_ (m^2^ s^–1^)	K_ma_ (1)	K^2^_ma_ D_m_ (m^2^ s^–1^)	Reference
Gypsum board				
Ethylbenzene	2.15 × 10^–11^	1550	5.17 × 10^–05^	Yang et al.^44^
Benzaldehyde	3.93 × 10^–11^	10053	3.97 × 10^–03^	Yang et al.^44^
Dodecane	1.73 × 10^–12^	34895	2.11 × 10^–03^	Yang et al.^44^
Particle board				
TVOC	7.65 × 10^–11^	3289	8.28 × 10^–04^	Yang et al.^45^
Hexanal	7.65 × 10^–11^	3289	8.28 × 10^–04^	Yang et al.^45^
α-Pinene	1.20 × 10^–10^	5602	3.77 × 10^–03^	Yang et al.^45^
Formaldehyde	4.47 × 10^–10^	560	1.40 × 10^–04^	Wang et al.^42^
Formaldehyde	6.71 × 10^–10^	149	1.49 × 10^–05^	Wang et al.^43^
Formaldehyde	1.40 × 10^–09^	84.1	9.90 × 10^–06^	Wang et al.^43^
Formaldehyde	2.67 × 10^–08^	1510	6.09 × 10^–02^	Caron et al.^40^
Acetone	2.50 × 10^–09^	216	1.17 × 10^–04^	Caron et al.^40^
Acetylaldehyde	3.67 × 10^–09^	186	1.27 × 10^–04^	Caron et al.^40^
Propanal	2.17 × 10^–09^	24	1.25 × 10^–06^	Caron et al.^40^
Butanal	1.17 × 10^–09^	298	1.36 × 10^–04^	Caron et al.^40^
Pentanal	1.33 × 10^–09^	298	1.18 × 10^–04^	Caron et al.^40^
Hexanal	2.33 × 10^–09^	305	2.17 × 10^–04^	Caron et al.^40^
Terpenes	4.40 × 10^–08^	111	5.42 × 10^–04^	Caron et al.^40^
Solid wood furniture				
Toluene	7.86 × 10^–11^	7477	4.39 × 10^–03^	Wang et al.^46^
*p*-Xylene	4.34 × 10^–11^	11917	6.16 × 10^–03^	Wang et al.^46^
Formaldehyde	3.87 × 10^–10^	5038	9.82 × 10^–03^	Wang et al.^46^
Carpet				
Toluene	4.31 × 10^–11^	6171	1.64 × 10^–03^	Bodalal et al.^39^
Nonane	2.83 × 10^–11^	6216	1.09 × 10^–03^	Huang and Haghighat^32^

Wang et al.^41^ presented a comprehensive summary of these key parameters for individual VOCs across a wide range of materials. Formaldehyde in particle boards, medium density fiberboard, and solid wood furniture are the most studied VOC-material combinations. However, their experimentally determined values of *D*_m_ and *K*_ma_ span over three and 2 orders of magnitude, respectively, depending on the selected reference source. For example, formaldehyde in the particle board (Table 1) yields the highest value of *K*_ma_^2^*D*_m_ = 6.09 × 10^–2^.^40^

This value is 400 times larger than those reported by Wang et al.^42,43^ which are 1.40 × 10^–4^, 1.49 × 10^–5^, and 9.90 × 10^–6^. Formaldehyde diffusion in the particle board reported by Caron et al.^40^ is therefore considered an outlier and not used for calculating the ventilation threshold.

## Verification of the Analytical Model

Making assumptions and simplifications is always a crucial part of designing a new model, and there is always a balance between model feasibility and accuracy. Some simplifications can be motivated if they enhance usability even though they come at the expense of some model accuracy. In this section, we provide justifications for the key simplifications made in the model, namely, the omission of surface resistance, assumption of semi-infinite material, and consideration of specific initial conditions.

### Verification of the Assumption on Zero Surface Resistance

**Figure 5:**
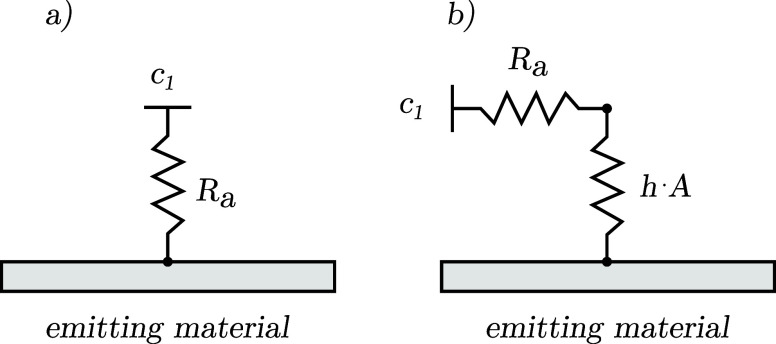
Circuit diagrams showing the mass transfer from the surface: (a) no surface resistance–emissions from the surface are directly linked to the outdoor air via ventilation, (b) surface convection is added between the surface and the ventilation air.

Assuming zero surface resistance implies that the concentration at the material surface is equal to that in nearby air. This situation is depicted by the left circuit diagram in Figure 5. Introducing surface resistance results in distinct concentrations at the material surface and in nearby air, illustrated by the addition of a new circuit in the diagram to the right.

With the convective mass transfer included the expression for *t*_c_ becomes
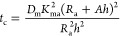
14where *h* (m s^–1^) is the convective mass transfer coefficient.

Values for *h* are not available in the literature to the same extent as diffusion and partition coefficients. Nevertheless, Huang and Haghighat^32^ provide data on diffusion, partition, and surface mass transfer coefficients for nonane emitted from a carpet. To evaluate the influence of surface resistance on the ventilation threshold, we used those data.

**Figure 6:**
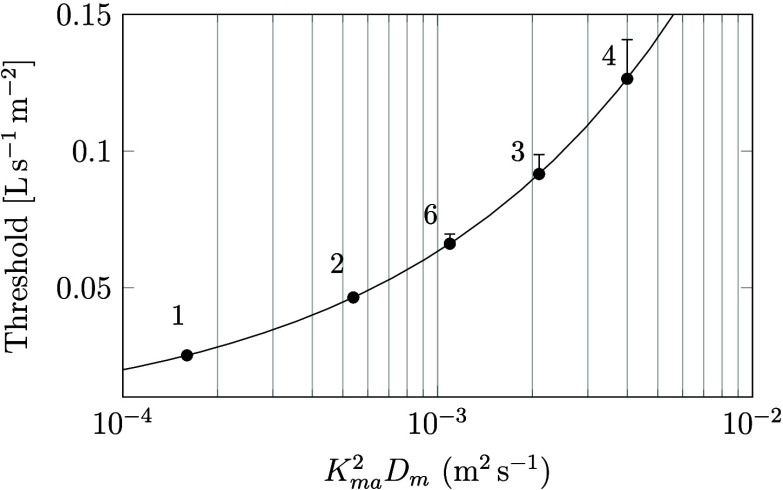
Ventilation thresholds calculated with eq 7 for *t*_c_^–0.5^ = 0.12 and plotted against *K*_m_^2^*D*_m_. Also, a selection of VOCs and materials (from Table 1) are marked in the graph: (1) toluene in carpet backing,^39^ (2) terpenes in particle board, (3) dodecane in gypsum board, (4) benzaldehyde in gypsum board, (6) nonane in carpet.^32^ The error bars show the ventilation threshold when convective mass transfer is included using eq 14 with *h* = 7.1 × 10^–4^ m s^–1^.

Surface mass transfer coefficients are provided for three air velocities, 0.01 m s^–1^ (*h* = 3.2 × 10^–4^ m s^–1^), 0.1 m s^–1^ (*h* = 7.1 × 10^–4^ m s^–1^), and 0.5 m s^–1^ (*h* = 1.0 × 10^–4^ m s^–1^). When using these data as input to eq 14, the resulting ventilation thresholds become, 0.075 L s^–1^ m^–2^ (0.01 m s^–1^), 0.070 L s^–1^ m^–2^ (0.1 m s^–1^), and 0.069 L s^–1^ m^–2^ (0.5 m s^–1^) while it is 0.066 L s^–1^ m^–2^ when there is no surface resistance. Thus, the ventilation threshold increases along with the surface resistance. The increase, compared to excluding surface resistance, is 13.8%, 5.5%, and 3.8% for each respective air velocity. Figure 6 shows a zoomed-in part of Figure 4 where the marked VOC-material combinations are complemented with error bars showing the ventilation threshold when the surface resistance corresponding to *h* = 7.1 × 10^–4^ m s^–1^ (air velocity of 0.1 m s^–1^) is included in eq 14. These are small errors compared to other uncertainties coming from the material data.

It is important to note that, in reality, the air velocity along the room’s surfaces depend on the ventilation rate and airflow pattern in the room. This is not accounted for in the above example, since air velocity and ventilation rate are not linked. However, the air velocities used in the example are within a range typically found in buildings, and the calculated errors can, therefore, serve as an approximation of the expected errors caused by excluding the surface resistance.

### Verification of the Assumption of Semi-infinite Material

In the comparison outlined in Table 2, determining the area of emitting surfaces posed challenges because the areas were not explicitly mentioned in the cited references. Finding the correct thickness of each emitting material is likely to be even more challenging. By assuming a semi-infinite material, we omit the need of defining material thickness. This assumption, in turn, results in some overestimation of the concentrations at the material surface over time as the semi-infinite material cannot be depleted. If the model was to be used for long-term predictions of VOC-concentrations in the indoor environment such an assumption would lead to overestimations of concentrations inside the material, especially at deeper levels.

**Table 2:** Summary of Comparison with Measurements^a^

*A* [m^2^]	ACR [h^–1^]	Threshold [h^–1^]	Outcome	Reference
164^b^	1.5	0.47	Unaffected	Noguchi et al.^48^
92^c^	0.8–1.7	0.19	Unaffected	Tuomainen et al.^49^
110	0.14–0.32	0.18	Affected	Hodgson et al.^23^
0.314	2.5–5.5	1.2	Unaffected^d^	Caron et al.^40^
0.314	2.5–5.5	4.5	Affected^e^	Caron et al.^40^

a *A* is the emitting area, ACR is the ventilation rates from the particular study, threshold is the calculated ventilation threshold at which increasing the ventilation will not affect the emission rate, and outcome specifies whether the emission rate was affected by change in ventilation rate in the study.

b The emitting area is estimated to be 2.5 times the floor area.

c The emitting area is assumed to be the same as the floor area.

d Only the emission rate for formaldehyde increased with 28%.

e Threshold calculated from material properties for formaldehyde.

**Figure 7:**
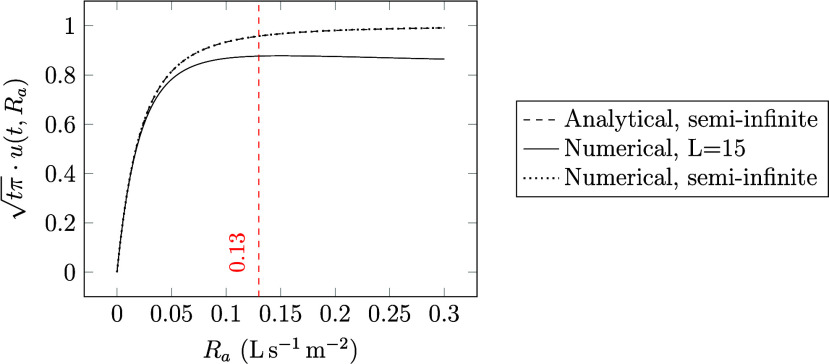
Comparison between semi-infite material and finite material using both analytical and numerical methods. The material used in the example is a 15 mm thick gypsum board and diffusion of benzaldehyde. Results are shown for different ventilation rates after 4 weeks.

To investigate the error caused by the assumption of a semi-infinite material and its effect on the ventilation threshold, some further comparisons are made using the diffusion of benzaldehyde in a 15 mm thick gypsum board as an example (material 2 in Table 1). The ventilation–emission relations for the finite and semi-infinite material are computed numerically using the Matlab programming environment and its *pdpe* function, specifically designed for solving initial-boundary value problems for systems of parabolic partial differential equations.^47^ Results from the calculations are listed in Figure 7.

From Figure 7 it is clear that the analytical and numerical solutions for the semi-infinite material are almost identical. Also all three solutions are nearly identical at lower ventilation rates.

All curves started to level out at similar ventilation rates. However, the curve describing the finite material has generally lower values because this model, in contrast to the semi-infinite models, accounts for the depletion of VOC inside the materials, which in turn affects the emission rate. Consequently, as time passes, the emission rate of these models will eventually reach zero. The semi-infinite material will, on the other hand, never get depleted and the corresponding solutions approach, therefore, nonzero values. Nevertheless, it is noteworthy that this discrepancy between the models remains relatively insignificant in the vicinity of the ventilation threshold point. Both curves start to level out at similar ventilation rates near the identified ventilation threshold, marked with a red vertical line. Since there is no major difference between the models in terms of identifying this threshold, both models can be used for this purpose. However, the semi-infinite model is advantageous since it requires fewer input data.

These results show that the assumption of a semi-infinite material has only minor influence on the shape of the curve in Figure 7, leading to small overestimations of the ventilation threshold. In other words, the ventilation threshold determined from a semi-infinite material can be regarded as an upper limit (conservative value).

### Verification of the Assumption of Initial Concentration

**Figure 8:**
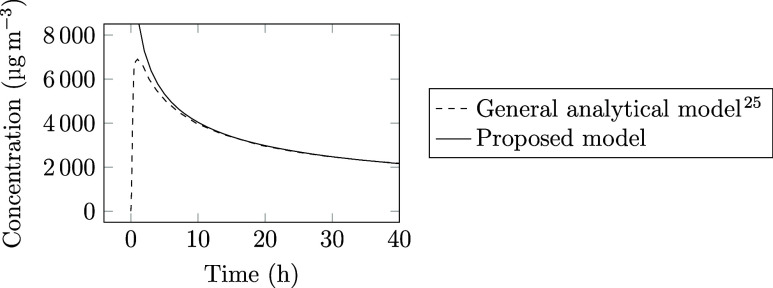
TVOC concentrations in a test chamber. Comparison between our model and the model proposed by Xiong et al.^25^

For simplicity reasons, the initial concentration in the room is assumed to be the same as that inside the material. To test the effect of this assumption a comparison is made with the general analytical model proposed by Xiong et al.^25^ Their model is validated with experimental data and accounts for several transport mechanisms, including different initial concentrations in air and the emitting material. Figure 8 shows a comparison between the detailed analytical model and our simplified model (eq 9), where the TVOC (total volatile organic compound) concentration in the air of a test chamber is modeled. The modeled material is a particle board, and the input parameters are *D*_m_ = 7.65 × 10^–11^, *c*_0_ = 5.28 × 10^7^, and *K*_ma_ = 3.289 × 10^3^.

In this comparison, it becomes clear that the assumption of having the same initial concentration in both the air and the material is affecting the results only at short times. Already after 10 h, both models show the same concentration in the air. Since we are typically interested in concentrations after a couple of weeks, the assumption of equal initial concentrations in the air and in the material is unlikely to significantly influence the results.

## Results: Application of the Ventilation Threshold

We will now apply the proposed ventilation threshold to compare results regarding the feasibility of enhanced ventilation, as reported in the literature. Results from Noguchi et al.,^48^ Tuomainen et al.,^49^ and Hodgson et al.^23^ are chosen for the comparison because they present data from field measurements where the emission rates are measured at different air change rates. Since none of these references provide data about emitting areas, assumptions are made based on the description of test buildings in each study. The assumptions are described along with the results from each comparison in Table 2. In addition, other results presented by Caron et al.,^40^ except the one for formaldehyde in particle board discussed further down, are also considered. These results are valuable as they include the emitting areas along with emission rates at different ventilation rates.

The earlier derived ventilation threshold of *R*_a_/*A* = 0.13 L s^–1^ m^–2^ is used in the calculations, corresponding to the highest *K*_ma_^2^*D*_m_ value in Table 2 (benzaldehyde). It is worth noting that this value represents an upper limit. By using the ventilation threshold and the estimated emitting area, air change rates (h^–1^) are found for each case as summarized in Table 2.

Noguchi et al.^48^ provide VOC emission rates in two identical rooms with different ventilation rates, expressed as air change rates. Because all surfaces were covered by wooden panels, we can assume that the emitting area is 2.5 times the floor area. By multiplying the ventilation threshold and the estimated emitting area, an air change rate of 0.47 h^–1^ is obtained. Air change rates in the study are 1.5 and 3.5 h^–1^. This means, according to our proposed method, that the reported ventilation rates have surpassed the threshold at which changes in ventilation have significant effects on emission rates. This is in line with the conclusion made by Hølos et al.^13^ when they reviewed the work of Noguchi et al.,^48^ which suggests that the relative reduction in VOC is similar in both rooms despite the differences in the ventilation rates. In other words, the emission rate is not affected by changes in the ventilation rate once they are above the ventilation threshold.

In Tuomainen et al.,^49^ two apartment buildings are compared, one with high-emitting materials and low ventilation rates and one with low-emitting materials and high ventilation rates. For this case, we assume that the emitting area is the same as the floor area. In their review of the results of Tuomainen et al.,^49^ Hølos^13^ assess that the same relative reduction in the emission rates can be observed in both buildings five months after the construction and concluded that the emission rates were not affected by the differences in ventilation rates. Our ventilation threshold gives an air change rate of 0.19 h^–1^ which is much lower than the actual ventilation rates in the buildings, 0.8 and 1.7 h^–1^. The emitting area in the calculation can be 4 times larger without the threshold surpassing the actual ventilation rates.

In contrast to the previous two studies, Hodgson et al.^23^ have found a correlation between the ventilation and TVOC emission rates in a newly built house for two different air change rates, 0.32 and 0.14 h^–1^. With the assumption that the emitting area is equal to the floor area, the ventilation threshold gives an air change rate of 0.18 h^–1^ which is between the two measured values. This means that a lower emission rate can be expected at 0.14 h^–1^ compared to 0.32 h^–1^ because the former is below the theoretical threshold. This is in line with the assessment in the review, where it is concluded that the emission rate for TVOC decreased by 30% at the lower ventilation rate.

In addition to the field studies in buildings provided earlier, we also compared the calculated and actual ventilation threshold using the results obtained from measurements in an experimental chamber conducted by Caron et al.^40^ Emissions of several VOCs from a wood particle board are measured at air change rates ranging from 2.5 to 5.5 h^–1^. When the ventilation rates were increased, no significant differences in the emission rates of the studied VOCs were observed, except for formaldehyde. With our method, the calucated ventilation threshold for the particle board is 1.2 h^–1^, which is lower than any ventilation rate used during the experiments, explaining why the emission rates are not affected by the increased ventilation.

However, the study reported a significant increase in the emission rates of formaldehyde (28%), when the ventilation was increased. The authors provided two possible explanations for this finding. First, they suggest that the hydrolysis of the binding resin in the particle board may be responsible for the excessive release of formaldehyde into the indoor environment. Additionally, in the chamber measurements, formaldehyde exhibits a much higher diffusivity compared to other VOCs. As mentioned earlier, the diffusion- and partition coefficients for formaldehyde determined in this study were much higher, *D*_m_ = 2.67 · 10^–8^ and *K*_ma_ = 1510, than in other literature, for example, *D*_m_ = 4.47 · 10^–10^ and *K*_ma_ = 560.^42^

Therefore, a new ventilation threshold is calculated based on the material properties for formaldehyde determined in the experiments, *K*_ma_^2^*D*_m_ ≈ 6.09 × 10^–2^. The new ventilation threshold per emitting area is 0.5 L s^–1^ m^–2^, corresponding to the air change rate of 4.5 h^–1^ in the chamber, which is higher than the lower air change rate (2.5 h^–1^) used in the experiment. Thus, our model correctly predicts that emission rates are affected by an increased ventilation rate.

Through these examples, we show that the proposed, physics-based ventilation threshold of 0.13 L s^–1^ m^–2^ (per square meter emitting area) is capable of signaling whether a further increase of ventilation rate will increase the emission rate and reduce the off-gassing time or not. Note, however, that somewhat different value can be obtained if the *t*_c_^–0.5^-threshold on 0.12 h^–0.5^ is chosen differently; see Figure 3.

## Discussion

A common strategy for dealing with initially high levels of VOCs in new buildings is to increase ventilation rates with the assumption that this will concurrently decrease indoor air concentrations and speed up the off-gassing of VOCs from materials. The ambition with our model is to explain, based on physics, that there is a ventilation threshold for an effective increase of emission rates, and that the established practice may lead to overventilation and unnecessary energy losses. There are several, more advanced analytical models, for example, Xiong et al.^25^ and Liu et al.,^26^ for modeling VOC emissions. Nonetheless, the distinctive advantage of our model lies in its emphasis on the interplay between emission and ventilation rates, with all model assumptions carefully tailored to this purpose. This is particularly evident in the curve depicted in Figure 3, which illustrates the connection between emission rate and the time constant (*t*_c_), a value derived from eq 7.

Another possible approach to determining a ventilation threshold is by comparing the total diffusion resistance within the material to the total convective mass transfer resistance at the material surface, including the convective boundary layer and the ventilation related resistance. For example, Xu and Zhang propose to use Biot numbers (*Bi*_m_) and Fourier numbers (*Fo*_m_) to determine a critical *Fo*_m,c_ at which the impact of *Bi*_m_/*K*_ma_ on the emission rate can be neglected.^50^ However, this approach needs two additional parameters, material thickness and convective mass transfer coefficient at the surface, *h*. Therefore, the advantage of our proposed method is its simplicity and dependence on relatively few model parameters.

The governing parameters for the ventilation threshold are the diffusion coefficient, *D*_m_, and the partition coefficient *K*_ma_ for a given combination of VOCs and materials. Higher values of *K*_ma_^2^*D*_m_ will lead to higher values of the area specific ventilation rates (L s^–1^ m^–2^). It means that for a specific material, the VOC with the highest value of *K*_ma_^2^*D*_m_ determines the ventilation threshold for the material.

The ventilation threshold can be expressed in other units, for example, air change rates per hour. However, the emitting areas are usually known (for example, area of gypsum board or carpet), and therefore, with an area specific ventilation rate, variations in emitting areas between different rooms are accounted for.

The simplification related to surface resistance between the material and the surrounding air is a significant aspect of our model. Here, the surface resistance is neglected while in reality the surface resistance exists and varies with the room’s ventilation pattern and rate. Although it is possible to account for variable surface transfer coefficients in eq 14, doing so necessitates an additional model parameter, which may not always be readily available. For example, one of the most comprehensive databases of diffusion coefficients, known to the authors, contains entries for about 120 different material VOC combinations.^41^ This can be compared with the Swedish database BASTA, which provides building products and information about their composition. Only in the category “plastic floor” there are nearly 8400 different products with varying compositions, materials, and colors. While the data extracted from literature provides valuable insights into the VOC-performance of typical building materials, it lacks validation to ensure its representativeness for materials found in the local market.^24^

As shown in Figure 6, this simplification introduces an error of up to 15% above the reference ventilation threshold, which is rather reasonable.

Similarly, the assumption of a well mixed concentration in the ventilated space may also influence the emission rate. If, for example, concentrations of VOC are found in higher quantities in some local area, this would influence the emission rate. However, for most cases, such as in the examples given in Table 2, the emitting areas make up a large portion of the total surface of the room and, therefore, the emission rate should be understood as a total rate for all the emitting surfaces. This means that local variations in the concentration are averaged out. However, to estimate the uncertainty caused by this assumption, a comparison can be made with the assumption of neglecting surface resistance at the material-air interface, as commented before.

With the proposed model for the ventilation threshold, conclusions from several field and laboratory measurements from the literature are benchmarked regarding the effects of increased ventilation on emission rates. For each case, a ventilation threshold is calculated based on the reported emission data, while in several cases assumptions about the emitting area are made. A full consistency between the predictions based on the ventilation threshold and the conclusions from the studies is found with a good margin. When the used ventilation rate is above the ventilation threshold, the conclusion from the cited work is that increased ventilation does not increase the emission rate and vice versa. This is a promising result regarding the model simplicity.

Another interesting observation refers to the actual values of the calculated thresholds for the field measurements, which are all below 0.5 h^–1^ (Table 2). As a comparison, the Swedish building code states that ventilation rates in residential buildings should be 0.35 L s^–1^ per square meter living area or higher which is about 0.5 h^–1^ at a normal ceiling height of 2.5 m.^51^ In addition, ventilation rates in offices and schools are often higher. For example, to handle nonoccupant related emissions, The Public Health Agency of Sweden recommends a ventilation rate of at least 0.35 L/s/m2 plus 7 L/s/person in schools and facilities for childcare.^52^

In addition, the ventilation thresholds in Table 2 were based on a material with a comparably high diffusion coefficient. Several materials have lower diffusion coefficients and will, therefore, yield a lower ventilation threshold. This result indicates that even ordinary ventilation has the capacity to remove higher concentrations of VOCs in new buildings in Sweden, which in turn opens possibilities for designing energy optimized ventilation schemes while preserving the good indoor comfort.

Also, it is important to acknowledge that temperature and humidity impacts the emission of organic compounds from materials through their influence on the diffusion and partition coefficient.^53^ For calculation of the ventilation threshold in an indoor environment with specific materials and hydrothermal conditions, diffusion and partition coefficients should be selected accordingly.

## Supporting Information Available

Detailed step by step derivation of the analytical model (PDF)

## Nomenclature

*A*: area of emitting material, m^2^
ACR: air change rate, h^–1^
*c*_a_: concentration in the air phase, μg m^–3^
*c*_m_: concentration in the material phase, μg m^–3^
*c*_0_: initial concentration, μg m^–3^
*c*_1_: concentration in supply air, μg m^–3^
*D*_m_: diffusion coefficient in the material, m^2^ s^–1^
*E*: emission rate, μg s^–1^
*h*: convective mass transfer coefficient at material-air interface, m s^–1^
*K*_*ma*_: material-air partition coefficient, 1
*R*_*a*_: ventilation rate, m^3^ s^–1^ or L s^–1^
*t*: time, s or h
*t*_c_: time constant, s or h
*x*: distance, m
