# Supplementary material for: Theoretical Threshold for Estimating the Impact of Ventilation on Materials’ Emissions
Source: Environ Sci Technol. 2024 Mar 6;58(11):5058–67. doi: 10.1021/acs.est.3c09815 (PMC10956430; doi:10.1021/acs.est.3c09815)
Supplement: Supplementary file 1 — es3c09815_si_001.pdf [file es3c09815_si_001.pdf]

Supporting information for: Theoretical threshold for estimating the impact of ventilation on materials' emissions

Fredrik Domhagen,[*,†] Sarka Langer,[†,‡] and Angela Sasic Kalagasidis[†]

†*Department of Architecture and Civil Engineering, Chalmers University of Technology, SE-41296 Gothenburg, Sweden*

‡*IVL Swedish Environmental Research Institute, P.O. Box 53021, SE-40014 Gothenburg, Sweden*

E-mail: fredrik.domhagen@chalmers.se

This PDF file includes:

Pages S1-S5

Emission from a semi-infinite material placed inside a ventilated space

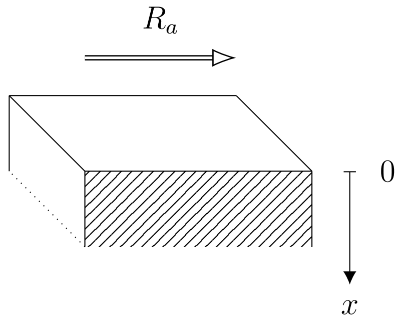

The governing equation for diffusion in the material:

$$D_m \frac{\partial^2 c_m}{\partial x^2} = \frac{\partial c_m}{\partial t} \quad \text{in} \quad 0 < x < \infty, \quad t > 0 \tag{1}$$

Equilibrium at the surface:

$$c_m(0,t) = K_{ma} \cdot c_a(0,t) \tag{2}$$

Boundary condition at the surface:

$$c_1 \frac{R_a}{A} = - D_m \frac{\partial c_m}{\partial x}\bigg|_{x=0} + c_a \frac{R_a}{A}\bigg|_{x=0} \tag{3}$$

$$= - D_m \frac{\partial c_m}{\partial x}\bigg|_{x=0} + \frac{c_m}{K_{ma}} \frac{R_a}{A}\bigg|_{x=0} \tag{4}$$

Boundary condition at infinity:

$$c_m\left(\infty, t\right) = c_0 \tag{5}$$

Initial condition:

$$c_m\left(x, 0\right) = c_0 \tag{6}$$

Laplace transformations:

$$\frac{d^2 \hat{c}_m(x,s)}{dx^2} - \frac{s}{D_m} \hat{c}_m(x,s) = - \frac{c_0}{D_m} \quad \text{in} \quad 0 < x < \infty \tag{7}$$

Boundary condition at the surface:

$$-D_m \frac{d\hat{c}_m}{dx}\bigg|_{x=0} + \frac{\hat{c}_m}{K_m}\frac{R_a}{A}\bigg|_{x=0} = \frac{1}{s}c_1\frac{R_a}{A} \tag{8}$$

Boundary condition at infinity:

$$\hat{c}_m(\infty, s) = \frac{c_0}{s} \tag{9}$$

Initial condition:

$$\hat{c}_m(x, 0) = \frac{c_0}{s} \tag{10}$$

The solution to the ordinary differential equation (Equation 7) is:

$$\hat{c}_m(x, s) = C_1 \exp\left(\sqrt{s/D_m}x\right) + C_2 \exp\left(-\sqrt{s/D_m}x\right) + \frac{c_0}{s} \tag{11}$$

Note that C_1 must be zero to ensure that the mass balance in Equation 9 is upheld. To find C_2 Equation 11 is differentiated:

$$\frac{d\hat{c}_m}{dx} = -C_2\sqrt{\frac{s}{D_m}}\exp\left(-\sqrt{s/D_m}x\right) \tag{12}$$

and plugged into the first boundary condition, Equation 8:

$$D_m\sqrt{\frac{s}{D_m}}C_2 + \left(C_2 + \frac{c_0}{s}\right)\frac{1}{K_{ma}}\frac{R_a}{A} = \frac{1}{s}c_1\frac{R_a}{A}$$

After some manipulation, the following expressions for C_2 and $\hat{c}_m$ are obtained:

$$C_2 = \frac{1}{s}\frac{R_a}{A}\frac{c_1 K_{ma} - c_0}{K_{ma}\sqrt{D_m}\sqrt{s} + \frac{R_a}{A}} \tag{13}$$

$$\hat{c}_m\left(x, s\right) = \frac{1}{s}\frac{R_a}{A}\frac{c_1 K_{ma} - c_0}{K_{ma}\sqrt{D_m}\sqrt{s} + \frac{R_a}{A}}\exp\left(-\sqrt{\frac{s}{D_m}}x\right) + \frac{c_0}{s} \tag{14}$$

The inverse Laplace transform of these expressions, translating them from the Laplace do-

main to the time domain, is found using the unilateral solutions available in the Laplace tables:

$$\hat{F}(s) = \frac{a \exp\left(-p\sqrt{s}\right)}{s\left(a+\sqrt{s}\right)} \quad p \geq 0 \tag{15}$$

$$\leftrightarrow$$

$$F(t) = -\exp\left(ap + a^2 t\right) \operatorname{erfc}\left(a\sqrt{t} + \frac{p}{2\sqrt{t}}\right) + \operatorname{erfc}\left(\frac{p}{2\sqrt{t}}\right) \tag{16}$$

by comparing Equation 14 with Equation 15 we find that:

$$p = \frac{x}{\sqrt{D_m}} \tag{17}$$

$$a = \frac{1}{K_{ma}} \frac{R_a}{A} \frac{1}{\sqrt{D_m}} \tag{18}$$

Plugging a and p into Equation 16, we get:

$$\begin{aligned} \frac{c_m(x,t) - c_0}{c_1 K_{ma} - c_0} &= \operatorname{erfc}\left(\frac{x}{2\sqrt{D_m t}}\right) \\ - \exp\left(\frac{1}{K_{ma}} \frac{R_a}{A} \frac{1}{\sqrt{D_m}} \frac{x}{\sqrt{D_m}} + \frac{1}{K_{ma}^2} \frac{R_a^2}{A^2} \frac{1}{D_m} t\right) \\ \cdot \operatorname{erfc}\left(\frac{1}{K_{ma}} \frac{R_a}{A} \frac{1}{\sqrt{D_m}} \sqrt{t} + \frac{x}{2\sqrt{D_m}\sqrt{t}}\right) + c_0 \end{aligned} \tag{19}$$

with:

$$t_c = \frac{K_{ma}^2 D_m A^2}{R_a^2} \tag{20}$$

the solution for the concentration of VOC within the material c(x,t) can be written:

$$\begin{aligned} \frac{c_m(x,t) - c_0}{c_1 K_{ma} - c_0} &= \operatorname{erfc}\left(\frac{x}{\sqrt{4 D_m t}}\right) \\ - \exp\left(\frac{x}{\sqrt{D_m t_c}} + \frac{t}{t_c}\right) \operatorname{erfc}\left(\sqrt{\frac{t}{t_c}} + \frac{x}{\sqrt{4 D_m t}}\right) \end{aligned} \tag{21}$$

and the flow of VOC out from the surface:

$$E = -AD_m \frac{\partial c}{\partial x} = (c_1 K_{ma} - c_0)\, A\sqrt{D_m}\, \mathrm{erfc}\left(\sqrt{\frac{t}{t_c}}\right) \exp\left(\frac{t}{t_c}\right) \frac{1}{\sqrt{t_c}} \tag{22}$$
